# Factors associated with visits to general practitioners in patients with schizophrenia in Malaga

**DOI:** 10.1186/s12875-018-0866-7

**Published:** 2018-11-28

**Authors:** M. C. Castillejos, C. Martín-Pérez, F. Mayoral-Cleries, A. Bordallo-Aragón, J. Sepúlveda-Muñoz, B. Moreno-Küstner

**Affiliations:** 1 Departament of Personality, Assessment and Psychological Treatment, University of Malaga, Campus Teatinos, Malaga, Spain; 2 Andalusian Group of Psychosocial Research (GAP), Malaga, Spain; 3 Andalusian Health Service, North East Granada Sanitary District, Clinical Management Unit at Marquesado, Carretera los Pozos, Alquife, Granada, Spain; 4 Clinical Management Unit of Mental Health of the Regional Hospital of Malaga, Andalusian Health Service, Paseo Limonar, Malaga, Spain; 5 Biomedical Research Institute of Malaga (IBIMA), Plaza del Hospital, Malaga, Spain; 6 Alameda-Perchel basic primary care team, Health District Malaga-Guadalhorce, Andalusian Health Service, Avenida Manuel Agustín Heredia, Malaga, Spain; 7 Departament of Personality, Assessment and Psychological Treatment, University of Malaga, Campus Teatinos, Malaga, Spain

**Keywords:** Primary health care, Schizophrenia, Use of services, Medical follow-up, Physical care

## Abstract

**Background:**

Patients with psychiatric disorders have more physical problems than other patients, so their follow-up by the general practitioner is particularly important for them.

**Methods:**

We aimed to elaborate a multilevel explanatory model of general practitioner (GP) visits made by patients with schizophrenia and related disorders (SRD). An observational, cross-sectional study was conducted from January 1, 2008 to July 1, 2011, in the area of the Clinical Management Unit of Mental Health (CMU-MH) of the Regional Hospital of Malaga (Spain). The eligible population consisted of all patients with SRD in contact with a GP residing in the study area. Our dependent variable was total number GP visits. The independent variables were: 1) patient variables (sociodemographic and clinical variables); 2) primary care centre (PCC) variables. We performed descriptive analysis, bivariate analysis and multilevel regression.

**Results:**

Four hundred ninety four patients were included. Mean annual number of GP visits was 4.1. Female sex, living in a socioeconomically deprived area, a diagnosis of schizoaffective disorder and contact with a GP who had a more active approach to mental health issues were associated with a higher number of visits whilst being single and good communication between the PCC and mental health teams were associated with a lower number of GP visits.

**Conclusions:**

Number of GP visits was not just associated with patient factors, but also with organisational and the involvement of health professionals, for example GPs with an active approach to mental health issues.

**Electronic supplementary material:**

The online version of this article (10.1186/s12875-018-0866-7) contains supplementary material, which is available to authorized users.

## Background

It has long been known that patients with severe mental disorders, such as schizophrenia or related disorders (SRD), have more physical health problems and higher mortality rates than the general population [1, 2] . Patients with SRD have a life expectancy 13–30 years shorter than the general population [3]. SRD have been associated with an increase in morbidity and mortality [4–6].

One of the reasons why the medical situation of these patients is worse is their inadequate approach to seeking health care when they require it [1, 7]. In addition, there is evidence that they make less use of primary care services [4, 8] and specialist services [9]. There is a risk that their physical co-morbidities are not detected by their GP [2, 10], so there is a greater risk that their physical health problems will be under-treated [11]. In some countries, such as Australia, training programs for doctors and nurses tend to minimise the importance of physical health care in mental health patients [12]. It has also been shown that patients with SRD benefit less from primary health care prevention and promotion initiatives than the general population [13, 14], despite the evidence that as a population they are more vulnerable to physical illnesses than the general population. It has been shown that regular treatment of patients with SRD in primary care improves survival [15]. It has also been shown that coordination between primary and specialist care is poor [16], which has a detrimental effect on the primary care of these patients.

Despite the above evidence, there have not been many studies analysing the use of primary care services by patients with SRD. The objectives of this article are to describe a multilevel explanatory model of GP visits by patients with SRD, to identify the patient factors and organisational, i.e. primary care centre (PCC) factors that are associated with number of GP visits.

## Methods

This was a descriptive, cross-sectional study.

### Study area and temporal scope

This study was carried out in the catchment area of the Clinical Management Unit of Mental Health (CMU-MH) of the Regional Hospital of Malaga (Spain) which has a population of 315,159. The area has two community mental health units (CMHU): Centre and North, associated with 13 PCCs.

The information on GP visits covers the 42-month period from January 1, 2008 to July 1, 2011.

### Eligible population and sample

A case register of patients with a diagnosis of SRD in the CMU-MH area of the Regional Hospital of Malaga, the Malaga Schizophrenia Case Register (RESMA), was developed to improve the care of patients with severe mental illness. Further information on the register is available in Moreno-Küstner et al. [17].

The population eligible for the study consisted of all patients on the RESMA (*N* = 1663) [18]. The sample size was calculated to be representative of the patients of each PCC. Patients were selected by simple random sampling, stratified by PCC.

The inclusion criteria were the following: a) age over 14 years old; b) clinical diagnosis of SRD according to the ICD-10; c) in contact with a PCC in the catchment area of the CMU-MH of the Regional Hospital of Malaga. The exclusion criteria were the following: a) receipt of treatment outside the study area; b) death during the observation period; c) no computerised medical history at the reference PCC.

### Study variables

Our dependent variable was total number of GP visits made by patients with SRD during the three and a half-year observation period.

The independent variables were grouped into two levels: 1) patient variables; 2) PCC variables.

Patient variables were divided into sociodemographic and clinical variables (Additional file 1: Table S1). PCC variables are also shown in Additional file 1: Table S1.

### Data sources

Data on total number of GP visits were obtained from digitalised primary care records (DIRAYA program). Sociodemographic and clinical information about patients was obtained from RESMA [17, 18]. Information about the PCCs was collected from their directors, who were interviewed using a questionnaire designed by the research team.

### Data analysis

The categorical variables used in the descriptive analysis were frequency distribution and percentages. Descriptive statistics (mean, standard deviation, median and quantiles) were calculated for the continuous variables.

We used bivariate analysis to examine the relationships between the dependent variable (number of GP visits) and the independent variables. The means of independent, dichotomous categorical variables were compared using the Wilcoxon test. The means of other independent categorical variables were compared using the Kruskal-Wallis test. Differences were considered significant at *p* < 0.05.

Finally, we constructed a multilevel, linear regression model in which level 1 was the patient, and level 2 the PCC. Beforehand we verified that there were differences between PCCs that allowed the application of the multilevel model. After all the variables were reintroduced into the model we removed non-significant variables step by step until a definitive model was obtained.

The statistical package R was used for the statistical analysis.

## Results

We discovered that 34 of the initial sample of 528 patients (6%) did not have a digitalised primary care history, so these patients were excluded from the study. Thirty-six of the final sample of 494 patients did not have any contact with their GP during the observation period.

The sociodemographic profile of the sample was as follows: 66.3% male; mean age 43.78 years old (standard deviation 11.64; range 19–83); 70.6% single; 64.4% had not completed secondary school; 56.9% living with their parents or friends; 42.9% unable to work; 87.1% living in an urban area and 11.5% in a socioeconomically deprived area.

Schizophrenia was the most frequent diagnosis (70%) (Table 1). The overall severity level had been evaluated in 431 cases (87.2%) and the majority were placed in the less severe classes (1 and 2) (Table 1).

**Table 1:** The sociodemographic and clinical characteristics of patients with schizophrenia and related disorders who were in contact with general practitioner. (*N* = 494)

Sociodemographic characteristics	Patients *N* (%)
Gender	
Male	327 (66.3%)
Female	167 (33.8%)
Age	
15–44	265 (53.6%)
45–64	205 (41.5%)
> 65	24 (4.9%)
Marital status	
Single	349 (70.6%)
Married/Civil partnership/Cohabiting	90 (18.2%)
Separated/Divorced/Widowed	55 (11.1%)
Educational level	
No formal education and/or illiterate	81 (16.4%)
Primary school	237 (48%)
Secondary school	126 (25.5%)
Higher education (Bachelor’s degree)	50 (10.1%)
Living arrangements	
Alone	54 (10.9%)
Original family/other relatives or friends	281 (56.9%)
Own family	102 (20.6%)
Sheltered accommodation	52 (10.5%)
Homeless	5 (1.0%)
Employment status	
Employed	76 (15.4%)
Unemployed	85 (17.2%)
Student	29 (5.9%)
Carer or househusband/housewife	30 (6.1%)
Not working, receiving welfare benefits	212 (42.9%)
Other	62 (12.6%)
Area	
Urban	430 (87.1%)
Rural	64 (12.9%)
Within a socioeconomically deprived area	
No	437 (88.5%)
Yes	57 (11.5%)
Primary care centre	
Trinidad	50 (10.1%)
Nueva Málaga	23 (4.7%)
Miraflores	18 (3.6%)
Palma-Palmilla	36 (7.3%)
Ciudad Jardín	45 (9.1%)
Capuchinos	32 (6.5%)
Carlinda	17 (3.4%)
Alameda Perchel	33 (6.7%)
Victoria	40 (8.1%)
Limonar	37 (7.5%)
El Palo	69 (14%)
Rincón de la Victoria	56 (11.3%)
Colmenar	2 (0.4%)
Out of the study area	36 (7.3)
(Missing data: 13)	
Community mental health centre	
Centre	272 (55.1%)
North	222 (44.9%)
Clinical characteristics	
ICD-10 Clinical diagnosis	
F20 Schizophrenia	346 (70%)
F22 Persistent delusional disorders	53 (10.7%)
F23 Acute and transient psychotic disorders	46 (7.3%)
F25 Schizoaffective disorders	36 (9.3%)
F21, F24, F28, F29 Schizotypal disorder, Induced delusional disorder, other non-organic psychotic disorders and unspecified non-organic psychosis	13 (2.6%)
Global level of severity	
Level I (low severity)	157 (31.8%)
Level II	189 (38.2%)
Level III (high severity)	85 (17.2%)
(Missing data:63)	
Total	494 (100%)

In 69.2% of the PCCs the mental health team visited 3 or more times per month, and in 30.8% of PCCs the team made a training visit once a month. Communication between the mental health team and GPs was rated as good or very good in 92.4% of cases, communication between GPs and primary care nurses was rated as good 84.6% of cases and communication between the social worker and GPs was rated as good in 46.2% of cases. When PCC directors were asked whether primary care professionals should play an active role in management of mental health problems 46.2% were neutral about the role of GPS and 53.8% were neutral about the role of primary care nurses. Finally, 69.3% of PCC directors agreed or totally agreed that social workers have an active role in management of mental health problems (Table 2).

**Table 2:** Organisation of the primary care centres

	Primary care centres N (%)
Social worker	
Shared	7 (53.8%)
Full time	6 (46.2%)
Primary care physicians play an active role in managing patients’ mental health	
Neither agree nor disagree	6 (46.2%)
Agree	5 (38.5%)
Completely agree	2 (15.4%)
Frequency of mental health care visits in primary care centres	
Twice a month	4 (30.8%)
More than three times a month	9 (69.2%)
Frequency of mental health training sessions in primary care centres	
None	3 (23.1%)
Once a year or less	1 (7.7%)
Between 4 and 6 months	1 (7.7%)
Every 2 months	2 (15.4%)
Once a month	4 (30.8%)
Twice a month	1 (7.7%)
More than three times a month	1 (7.7%)
How would you rate the communications between the primary care centre and the community mental health centre?	
Neither good nor bad	1 (7.7%)
Good	6 (46.2%)
Very good	6 (46.2%)
How would you rate the communications between primary care physicians and nurses?	
Good	11 (84.6%)
Very good	2 (15.4%)
Nurses play an active role in managing patients’ mental health	
Completely disagree	1 (7.7%)
Neither agree nor disagree	7 (53.8%)
Agree	2 (15.4%)
Completely agree	3 (23.1%)
How do you rate the level of communication between primary care physicians and social workers?	
Not applicable	1 (7.7%)
Neither good nor bad	3 (23.1%)
Good	6 (46.2%)
Very good	3 (23.1%)
Social workers play an active role in managing patients’ mental health	
Not applicable	1 (7.7%)
Disagree	1 (7.7%)
Neither agree nor disagree	2 (15.4%)
Agree	4 (30.8%)
Completely agree	5 (38.5%)
Total	13 (100%)

In total there were 7087 GP visits during the 42-month observation period (annual mean = 4.1 visits per patient, 42-month mean = 14.35 visits per patient, 42-month SD (Standard Deviation) = 12.44, range = 0–75).

Results of the bivariate analysis are shown in Tables 3 and 4.The full multilevel, linear regression model explained 17.5% of the estimated variance in GP visits. The patient variables that were independently positively associated with number of GP visits were: female sex (*p* < 0.001); being married/living with partner (*p* = 0.005), being separated/divorced/widowed (*p* = 0.048); living in a socioeconomically deprived area (*p* = 0.002). The following patient variables were associated with fewer GP visits relative to diagnosis of schizoaffective disorder: diagnosis of schizophrenia (*p* = 0.005), persistent delusional disorders (*p* = 0.05), acute and transient psychotic disorders (*p* = 0.004) and schizotypal disorder, induced delusional disorder, other non-organic psychotic disorder or unspecified non-organic psychosis (*p* = 0.012). With regard to PCC variables, having a GP who took a more active approach to mental health issues was positively associated with number of GP visits. However, good or very good communication between the mental health team and primary care professionals was associated with fewer GP visits (Table 5).

**Table 3:** Bivariate analysis between dichotomous variables and number of contact with general practitioner

	Median	W Wilconxon	*P* valor
Area		17,262	0.001
Urban	12		
Rural	7.5		
Mental health area		27,869	0.1409
North	11		
Centre	13		
Socioeconomic deprived area		9010.5	0.000
Yes	11		
No	16		
Gender		35,144	0.000
Male	17		
Female	11

**Table 4:** Bivariate analysis between categorical variables and number of contact with general practitioner

	Krukal-Wallis Chi cuadrado	d.f.	*P* value
Age (3 groups)	1.12	2	0.572
Marital status	12.29	2	0.002
Educational level	2.35	3	0.504
Living arrangements	6.21	4	0.184
Employment status	2.97	5	0.705
Primary care centre	40.93	16	0.000
ICD-10 clinical diagnosis	10.35	4	0.035
Primary care physicians play an active role in managing patients’ mental health	11.95	2	0.003
Frequency of visits to primary health care centres to manage mental health	1.19	1	0.276
Frequency of mental health training sessions in primary healthcare centres	35.91	6	0.000
How would you rate the communications between the primary care centre and the community mental health centre?	6.63	2	0.036
How would you rate the communications between primary care physicians and nurses?	4.58	1	0.032
Nurses play an active role in managing patients’ mental health	7.85	3	0.005
How would you rate the communications between primary care physicians and social workers?	6.90	3	0.075
Social workers play an active role in managing patients’ mental health	13.62	4	0.009

**Table 5:** Multilevel linear regression of number of contact of patient with Schizophrenia and related disorders, N = 494

	Coefficient	*P* value
Gender		
Women	1	
Men	−5.783	0.000
Area		
Urban	1	
Rural	3.496	0.0939
Marital status		
Single	1	
Married/Civil partnership/Cohabiting	4.258	0.0051
Separated/Divorced/Widowed	3.518	0.0486
ICD-10 clinical diagnosis		
F25	1	
F20	−10.825	0.0051
F22	−5.299	0.0494
F23	−7.902	0.0035
F21, F24, F28, F29	−5.481	0.0124
Socioeconomically deprived area		
No	1	
Yes	5.759	0.0019
Primary care physicians play an active role in managing patients’ mental health		
Neither agree nor disagree	1	
Agree	−1.333	0.4229
Completely agree	3.925	0.0254
How would you rate the communications between the primary care centre and the community mental health centre?	1	
Neither good nor bad	−5.314	0.0121
Good	−4.801	0.0133
Very good		

## Discussion

This study used multilevel linear regression to examine associations between number of GP visits and patient and organisational factors. Some patient variables - female sex, having been married or cohabiting at some point and a diagnosis of schizoaffective disorder - were positively associated with number of GP visits. Another important finding was that having a GP who took a more active approach to mental health issues was positively associated with number of GP visits, whereas the presence of good or very good care communication between the PCC and community mental health professionals was negatively associated with number of GP visits.

The mean annual number of patient contacts with GP was 4.1, which is in line with other reports [5, 16, 19]. Our results show that in the area we studied the majority of patients were in contact with primary care professionals, only 3.2% had no contact with a GP during the observation period, which is even lower than in a study conducted in Norway, in which the percentage of patients who had no contacts with a GP was 17% [19].

The multilevel linear regression showed that women made more visits to their GP, which is in line with other studies [4, 15, 19]. The analysis also showed that patients who were married or living with a partner had more contact with PPCs than singles, as in an earlier study [15] and this may have been because family support helped to ensure they received continuing care. However, we also found that patients who were separated, divorced or widowed also visited their GP more frequently than singles, in contrast to the aforementioned study [15]. Number of GP visits was also positively associated with a diagnosis of schizoaffective disorder. We have not found any other study that analysed the relationship between GP visits and form of psychotic disorder. Living in a socioeconomically deprive areas was positively associated with number of GP visits, which is consistent with a previous study [20]. This may be because in areas of socioeconomic deprivation the incidence of SRD is greater [21].

We found that GPs who took a more active approach to mental health issues were visited more frequently by patients, which implies a better medical follow-up of the patient. However communication between PCC and CMHCs was negatively associated with number of GP visits. This may be because good communication between the team resulted in community mental health professionals assuming some of the functions that would otherwise have been performed by the GP, such as monitoring physical health problems, and this could be because GPs feel unprepared and with low confidence in treating patients with severe mental health problems [22, 23], even if it is about their physical health problems. In the CMHU of the Regional Hospital in Malaga specialist mental health care has been coordinated with primary care for more than 20 years. This coordination means that once a week PCCs are visited by a specialist mental health professional who will deliver care to patients with less severe mental illness. In addition, mental health and primary care professionals hold joint meetings about patients. We found that mental health team visits to PCCs usually occurred 3 or more times a month and training visits took place once a month. In 92.4% of cases communication between the CMHU and GP was rated at as good or very good; in a similar study carried out in Catalonia cooperation between primary care and specialist services in mental health was considered satisfactory [24]. These results contrast with a report that French GPs thought there was a lack of communication between mental health services and primary care services [25]. The importance of collaboration between primary care and specialist mental health services has been shown in several studies. Good communication is associated with greater GP satisfaction with specialist services, shorter referral times, shorter treatment duration, fewer appointments and lower treatment costs [26]. Although GPs feel responsible for management of physical or mental health in schizophrenia and are willing to work with mental health teams [10] and early interventions [27], they have yet to embrace it. A robust system of collaboration between levels of care would be necessary to achieve this [28–30].

### Strengths and limitations

A strength of this study is that we analysed a large, homogeneous sample of patients with a diagnosis of SRD living in the community and have thus provided an unbiased picture of GP visits by this population. We also want to point out that we have used multilevel linear regression with two levels, patient and PPC.

As to limitations, this was a cross-sectional study so we cannot infer causality, only association. In addition diagnoses were not based on structured diagnostic interviews, but they were clinical diagnoses made by the patients’ long-term psychiatrists and updated in the RESMA database. The data were obtained from databases that are routinely filled in clinical practice, so we cannot their reliability. Finally, it would have been interesting to have data on a matched control population.

## Conclusions

Our results reveals that the number of GP visits made by patients with SRD is affected by organisational factors, such as the GP’s approach to mental health issues, as well as patients factors.

Medical follow-up of the physical illness is crucial in patients with SRD, and primary care services are central in it. More studies are needed in this field; in this way we improve awareness of the need for GPs’ involvement in managing the physical health of patients with SRD.

## Additional file

Additional file 1 **Table S1.** Study variables. (DOCX 15 kb)
