# Supplementary material for: Factors associated with visits to general practitioners in patients with schizophrenia in Malaga
Source: BMC Fam Pract. 2018 Nov 28;19:180. doi: 10.1186/s12875-018-0866-7 (PMC6264610; doi:10.1186/s12875-018-0866-7)
Supplement: Supplementary file 1 — Table S1. Study variables. (DOCX 15 kb) [file 12875_2018_866_MOESM1_ESM.docx]

**TABLE S1**: Study variables.

| **PATIENT VARIABLES** | |
| --- | --- |
| **Sociodemographic variables** | **Categories** |
| **Gender** | Male  Female |
| **Age** | 15-44  45-64  >65 |
| **Marital status** | Single  Married/Civil partnership/Cohabiting  Separated/Divorced/Widowed |
| **Educational level** | No formal education and/or illiterate  Primary school  Secondary school  Higher education (Bachelor’s degree) |
| Living arrangements | Alone  Original family / other relatives or friends  Own family  Sheltered accommodation  Homeless |
| **Employment status** | Employed  Unemployed  Student  Carer or househusband/housewife  Not working, receiving welfare benefits  Other |
| **Area** | Urban  Rural |
| **Within a socioeconomically deprived area^1^** | No  Yes |
| **Primary care centre** | Trinidad  Nueva Málaga  Miraflores  Palma-Palmilla  Ciudad Jardín  Capuchinos  Carlinda  Alameda Perchel  Victoria  Limonar  El Palo  Rincón de la Victoria  Colmenar  Outside the study area |
| **Community mental health centre** | Centre  North |
| **Clinical variables** | |
| **ICD-10 clinical diagnosis** | F20 Schizophrenia  F22 Persistent delusional disorders  F23 Acute and transient psychotic disorders  F25 Schizoaffective disorders  F21, F24, F28, F29 Schizotypal disorder, **Induced delusional disorder,** other non-organic psychotic disorders and unspecified non-organic psychosis |
| **Global level of severity** | Level I (low severity)  Level II  Level III (high severity) |
| **PRIMARY CARE CENTRE VARIABLES** | |
| **Social Worker** | None  Shared  Full time |
| **Primary care physicians play an active role in managing patients’ mental health** | Completely disagree  Disagree  Neither agree nor disagree  Agree  Completely agree |
| **Frequency of mental health care visits in primary care centres** | None  Once a year or less  Between 4 and 6 months  Every 3 months  Every 2 months  Once a month  Twice a month  More than three times a month |
| **Frequency of mental health training sessions in primary care centres** | None  Once a year or less  Between 4 and 6 months  Every 3 months  Every 2 months  Once a month  Twice a month  More than three times a month |
| **How would you rate the communications between the primary care centre and community mental health centre?** | Very bad  Bad  Neither good nor bad  Good  Very good |
| **How would you rate the communications of the centre’s primary care physicians and/or nurses?** | Very bad  Bad  Neither good nor bad  Good  Very good |
| **Nurses play an active role in managing patients’ mental health** | Completely disagree  Disagree  Neither agree nor disagree  Agree  Completely agree |
| **How would you rate the level of communication between primary care physicians and social workers?** | Not applicable  Very bad  Bad  Neither good nor bad  Good  Very good |
| Social workers play an active role in managing patients’ mental health | Not applicable  Completely disagree  Disagree  Neither agree nor disagree  Agree  Completely agree |
| ^1T^The patient’s residence was considered to be in a socioeconomically deprived area (yes/no) according to the classification of the Andalusian Goverment (Junta de Andalucía, 2004). | |
